# Association among attention-deficit hyperactivity disorder, restless legs syndrome, and peripheral iron status: a two-sample Mendelian randomization study

**DOI:** 10.3389/fpsyt.2024.1310259

**Published:** 2024-05-08

**Authors:** Guoqiang Xiao, Hongting Shi, Qiaoyu Lan, Jiajia Hu, Jincheng Guan, Zhuoji Liang, Chumeng Zhou, Zitong Huang, Yongyuan Chen, Borong Zhou

**Affiliations:** ^1^ Department of Psychiatry and Psychology, Guangdong Provincial Key Laboratory of Major Obstetric Diseases, Guangdong Provincial Clinical Research Center for Obstetrics and Gynecology, The Third Affiliated Hospital of Guangzhou Medical University, Guangzhou, China; ^2^ Department of Neurology, The Second Affiliated Hospital of Guangzhou Medical University, Guangzhou, China; ^3^ Department of Neurology, Longhua District People’s Hospital, Shenzhen, China; ^4^ Department of Neurology, Guangdong Provincial Key Laboratory of Major Obstetric Diseases, Guangdong Provincial Clinical Research Center for Obstetrics and Gynecology, The Third Affiliated Hospital of Guangzhou Medical University, Guangzhou, China; ^5^ Medical Administration College, Guangzhou Medical University, Guangzhou, China; ^6^ Department of Neurology, The Fifth Affiliated Hospital of Guangzhou Medical University, Guangzhou, China

**Keywords:** attention deficit hyperactivity disorder, ADHD, restless legs syndrome, RLS, iron status, MR, LDSC

## Abstract

**Background:**

Epidemiological evidence indicates a high correlation and comorbidity between Attention Deficit Hyperactivity Disorder (ADHD) and Restless Legs Syndrome (RLS).

**Objective:**

We aimed to investigate the causal relationship and shared genetic architecture between ADHD and RLS, as well as explore potential causal associations between both disorders and peripheral iron status.

**Methods:**

We performed two-sample Mendelian randomization (MR) analyses using summary statistics from genome-wide meta-analyses of ADHD, RLS, and peripheral iron status (serum iron, ferritin, transferrin saturation, and total iron binding capacity). Additionally, we employed linkage disequilibrium score regression (LDSC) to assess genetic correlations between ADHD and RLS using genetic data.

**Results:**

Our MR results supports a causal effect from ADHD (as exposure) to RLS (as outcome) (inverse variance weighted OR = 1.20, 95% CI: 1.08-1.34, p = 0.001). Conversely, we found no a causal association from RLS to ADHD (inverse variance weighted OR = 1.04, 95% CI: 0.99-1.09, p = 0.11). LDSC analysis did not detect a significant genetic correlation between RLS and ADHD (Rg = 0.3, SE = 0.16, p = 0.068). Furthermore, no evidence supported a causal relationship between peripheral iron deficiency and the RLS or ADHD onset. However, RLS may have been associated with a genetic predisposition to reduced serum ferritin levels (OR = 1.20, 95% CI: 1.00-1.04, p = 0.047).

**Conclusion:**

This study suggests that ADHD is an independent risk factor for RLS, while RLS may confer a genetic predisposition to reduced serum ferritin levels.

**Limitations:**

The GWAS summary data utilized originated from populations of European ancestry, limiting the generalizability of conclusions to other populations.

**Clinical implications:**

The potential co-occurrence of RLS in individuals with ADHD should be considered during diagnosis and treatment. Moreover, iron supplementation may be beneficial for alleviating RLS symptoms.

## 1 Introduction

Attention Deficit Hyperactivity Disorder (ADHD) is a common neurodevelopmental disorder characterized by age-inappropriate attention deficits, hyperactivity, and impulsivity (1). The global prevalence of ADHD is estimated to be between 5.29% and 7.2% (2–4), placing a heavy burden on society (5). Compared to healthy individuals, ADHD patients are more likely to experience alcohol abuse, mental disorders, criminal behavior, and antisocial personality, among other adverse outcomes (6). A recent joint survey by the World Health Organization and the International Association for the Study of Attention Deficit Hyperactivity Disorder (ADHD) released in 2022 showed that approximately 15.9% of college students have ADHD, with 58.4% having comorbidities, and this number is still on the rise (7). Genetic factors, exposure to toxins (8), perinatal factors, and nutritional deficiencies are recognized as risk factors for ADHD (9). The relationship between restless legs syndrome (RLS) and ADHD has received great attention, but to date, the pathogenesis remains unclear.

Restless Legs Syndrome (RLS) is a sleep disorder-related neurological disease. A European epidemiological survey showed that the prevalence of RLS in Europe has reached around 5%-10% and is still on the rise (10). Among children, the prevalence is about 2% (11), which may be greatly underestimated due to the difficulty of describing symptoms in children by themselves. Previous studies have shown that up to 44% percent of patients with Attention Deficit Hyperactivity Disorder have comorbid RLS, and 26%-33% percent of RLS patients have been diagnosed with ADHD or ADHD symptoms (12, 13). A meta-analysis showed that among subjects meeting RLS criteria, 3.2-17.4% percent had ADHD. In the ADHD group, RLS symptoms were present in 11%-42.9% percent of children and 20-33% of adults (14). Another case-control study also expressed the same view (15).These findings all suggest a steady comorbidity between RLS and ADHD. Despite extensive research into the pathophysiology of ADHD and RLS, the existence of a causal relationship between the two remains a subject of debate.

At the same time, a large number of clinical evidences suggest that iron supplementation can greatly alleviate the symptoms of ADHD and RLS (16), suggesting that the pathogenesis of both may be closely related to iron deficiency (17), but the role of iron deficiency in the pathogenesis of both remains unclear.

Randomized controlled trials (RCTs) are the gold standard for establishing causal relationships between diseases and interventions, but they can be expensive and time-consuming. Mendelian randomization (MR) is an alternative method for exploring potential causal associations between specific exposures and outcomes based on genome-wide association studies (GWAS) by simulating the random segregation and recombination of gametes (18). The principle is to treat genetic variation as a natural experiment (genetic variation is relatively less affected by measurement error or bias), where individuals are randomly assigned to different exposure levels, and genetic variation is used as an instrumental variable (IV). The grouping basis for IV analysis is not whether or not an intervention is received, but rather grouping subjects based on the distribution of IVs, thereby maximizing correction for bias caused by measurable and unmeasurable confounding factors (19). That is, genetic variation is randomly assigned and therefore not affected by confounding factors. A systematic review revealed few psychiatric Mendelian randomization studies on ADHD comorbidities. Our study addressed this gap by integrating analysis of genetic factors and disease mechanisms to explore ADHD psychiatric comorbidity under Mendelian randomization (20).

Linkage disequilibrium score regression (LDSC) estimates the contributions of polygenic architecture and confounding factors (such as population stratification, cryptic relatedness, and batch effects) using GWAS summary statistics. This allows for the evaluation of the genetic correlation between different traits, providing insights into the shared genetic architecture and potential causal relationships (21).

In this study, we employed MR and LDSC analyses using GWAS meta-analyses of ADHD and RLS to investigate the causal influence of ADHD on RLS risk and assess their shared genetic architecture. Guided by clinical evidence, we further applied MR analyses to explore potential causal relationships between ADHD, RLS, and Peripheral iron status.

## 2 Methods

We used Two-sample MR and LDSC to investigate the causal relationship and genetic correlation between ADHD and RLS, and Two-sample MR to explore the causal relationship among Peripheral Iron status, ADHD, and RLS. All data were obtained from publicly shared databases, without the need for participant consent.

We employed two-sample Mendelian randomization (MR) and linkage disequilibrium score regression (LDSC) to investigate the causal influence of ADHD on RLS risk and their shared genetic architecture. Additional two-sample MR analyses explored potential causal relationships among Peripheral Iron status, ADHD, and RLS. All data were obtained from publicly shared databases, without the need for participant consent.

### 2.1 Data sources

The summary statistics for RLS-associated GWAS-identified SNPs from a previous meta-analysis (22) were used in this study, which comprised individuals of European ancestry (n = 10,257 cases, 470,725 controls) from six cohorts (Iceland, Denmark, UK INTERVAL, UK Biobank, US Emory, and the Netherlands). All cases were diagnosed according to the International RLS Study Group 2003 criteria or the Cambridge-Hopkins RLS questionnaire. (Details included in **Table 1**)

**Table 1:** Details of the GWASs included in the Mendelian randomization.

Consortium	Phenotype	Participants	Web Source
**A meta-analysis of GWAS**	Restless legs syndrome	480982	https://www.nature.com/articles/s42003-020-01430-1
**PGC**	Attention deficit and hyperactivity disorder	314677	https://pgc.unc.edu/for-researchers/download-results/
**A meta-analysis of GWAS**	Iron status	246139	https://www.nature.com/articles/s42003-020-01575-z

GWAS, Genome-Wide Association Studies; PGC, Psychiatric Genomics Consortium.

The summary statistics for ADHD were obtained from a Psychiatric Genomics Consortium (PGC) study (https://pgc.unc.edu/for-researchers/download-results/) (23), comprising 38,691 cases and 275,986 controls. Details about the GWAS and data are provided in the original research. (Details included in **Table 1**)

Iron status-associated SNPs were derived from a European ancestry GWAS by Bell et al. (2021) (24), comprising 246,139 individuals with separate data for serum iron (n=163,511), ferritin (n=246,139), transferrin saturation (n=131,471), and total iron binding capacity (n=135,430) from Iceland, UK, and Denmark. A meta-analysis of 17 GWASs adjusted for sex, age, ancestry, and principal components served as the source. (Details included in **Table 1**)

### 2.2 Genetic variation and instrumental variable selection

Mendelian randomization (MR) relies on three key assumptions for valid causal inference: (1) strong association between the instrument (IV) and the exposure, (2) no pleiotropy or confounding biases affecting the exposure-outcome relationship, and (3) non-directional influence of genetic variation on the outcome only through exposure (25). SNPs satisfying these criteria qualify as instrumental variables (IVs). We implemented stringent selection criteria for IVs, including: The P-value threshold for IV selection (P<5x10e-8), and LD proxies are defined using 1000 European genome sample data. To avoid double-counting the effect of specific causal variation, a stringent linkage disequilibrium (LD) threshold (R2 <0.001, linkage disequilibrium region width =10000 kb) was used, and further quality control was based on a minor allele frequency >1%. We also calculated F-statistics to ensure that all SNPs had F-statistics greater than 10, using the formula: F=(beta/se)^2, indicating that the likelihood of bias due to weak instrument variability is very small (26). Finally, 26 SNPs related to ADHD met the criteria for use as instrumental variables (IVs); 16 SNPs related to RLS met the criteria for use as instrumental variables (IVs); and SNPs related to iron status, including ferritin (62), iron (27), total iron binding capacity (27), and transferrin saturation (35), met the criteria for use as instrumental variables.

### 2.3 Statistical analysis

We performed two-sample Mendelian randomization (MR) analyses using the TwoSampleMR package (v0.5.6) in R to explore the causal relationships between ADHD, RLS, and Peripheral iron status. In harmonizing the effect allele of GWAS for exposure and outcome data, incompatible SNPs were removed, as well as palindromic SNPs with intermediate allele frequency, to ensure that the SNP effect on exposure corresponds to the effect allele of the palindromic SNP on the outcome: rs10188680, rs112716420, rs3112620, and rs996064. For SNPs related to RLS, 16 SNPs were used as instrumental variables (IVs), with palindromic SNPs: rs114142727, rs73145587, and rs76284431. SNPs related to iron status were also removed for incompatible and palindromic SNPs using the above method(SNPs used for MR analysis can be found in **Supplementary Materials**).

To address potential heterogeneity and outliers, we employed rigorous sensitivity analyses. Heterogeneous SNPs were iteratively removed using the leave-one-out method until Cochran’s Q statistic no longer indicated significant heterogeneity (details in **Supplementary Materials**). Additionally, outliers were identified and excluded using the Pleiotropy Residual Sum and Outlier (PRESSO) test. We employed three MR models (Inverse-Variance Weighted (IVW), MR-Egger, and Weighted Median) to assess the causal effect of RLS on ADHD, accounting for different assumptions about pleiotropy. These models adhere to the core principles of MR: relevance (IVs associated with exposure), independence (IVs unassociated with confounders), and exclusion (IVs influencing outcome only through exposure). IVW with random effects served as the primary MR analysis, providing an efficient estimate under the assumption of no pleiotropy. MR-Egger and Weighted Median, less sensitive to pleiotropic effects, served as supportive analyses. Consistent results across models strengthened the reliability of our findings. Finally, to control for potential false positives due to multiple tests, we applied the False Discovery Rate (FDR) correction to the primary IVW p-value (27).

### 2.4 Sensitivity analysis

**Figure 1:**
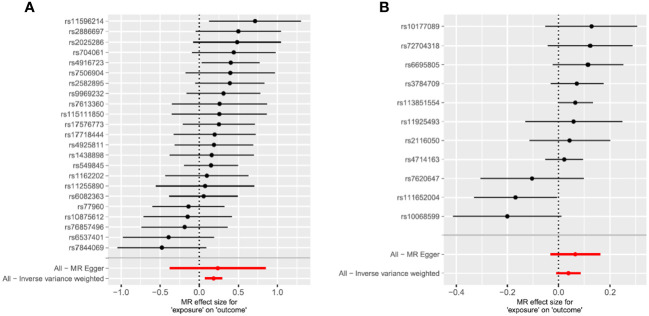
Mendelian randomization study results are displayed in forest plots to assess the potential causal relationship between ADHD and RLS. **(A)** The forest plot displays point estimates of ADHD exposure on RLS (outcome). After excluding pleiotropic, heterogeneous, incompatible, and palindromic SNPs, 23 index SNPs remained to construct instrument variables. **(B)** The forest plot displays point estimates of RLS exposure on ADHD (outcome). After excluding pleiotropic, heterogeneous, incompatible, and palindromic SNPs, 11 index SNPs remained to construct instrument variables. The black dots indicate the causal estimate (b = log odds ratio) of each SNP on the risk of ADHD. The red dots indicate the combined causal estimate of all SNPs using MR Egger and IVW methods. Horizontal lines represent 95% CI.

Sensitivity analysis is crucial in MR studies to detect whether potential pleiotropy and heterogeneity of MR estimates have been severely violated. We typically use the Cochran Q test to assess heterogeneity between SNP estimates. If the P-value of the Cochran Q test is <0.05, the result indicates the presence of heterogeneity, representing statistically significant heterogeneity between exposure and outcome, and we need to select the IVW random effects model as the gold standard. If there is no heterogeneity, both fixed and random effects models can be used as research results, and forest plots are used to visualize the results of heterogeneity testing. The Egger-intercept method and PRESSO test were also used to test whether the Mendelian randomization assumption was violated due to the presence of horizontal pleiotropy (28). If the intercept term in the MR-Egger intercept test analysis has a P-value <0.05, it indicates that the MR analysis has significant horizontal pleiotropy, suggesting that the related research is unreliable. MR-PRESSO consists of three parts: a) detection of horizontal pleiotropy; b) correction of horizontal pleiotropy by removing outliers; and c) testing for significant differences between causal estimates before and after removing outliers. We used PRESSO testing to remove outliers. The leave-one-out method was used to remove individual SNPs to assess whether MR estimates were driven or biased by individual SNPs. Finally, scatter plots, funnel plots, and forest plots were used for visualization(All plots generated from the analysis can be seen in **Figure 1** and **Supplementary Figure**. Through the above research tests, if the IVW test shows P<0.05, it suggests that there is a causal relationship between exposure and outcome in MR analysis.

### 2.5 Genetic correlation analyses

The genetic correlation between Attention Deficit Hyperactivity Disorder (ADHD) and Restless Legs Syndrome (RLS) was assessed utilizing LD score regression (LDSC) and Genome-wide association study (GWAS) data. GWAS summary statistics for ADHD and RLS were procured and single nucleotide polymorphisms (SNPs) were computed z scores standardized. The processed GWAS data were incorporated into the LDSC model, with each SNP’s LD score as the predictor and GWAS z-scores as the outcome, constructing a regression equation (21, 29). This regression model leverages linkage disequilibrium (LD) and GWAS statistics to estimate the genetic correlation between ADHD and RLS.

## 3 Results

Based on GWAS summary statistics and using research methods such as Two-sample MR and LDSC, we found that the genetic predisposition of ADHD may increase the risk of developing Restless Legs Syndrome (RLS), but there is no evidence to support a reverse causal relationship. At the same time, using LDSC, we found no genetic correlation between the two. We also used MR to analyze GWAS related to RLS, ADHD, and iron status, and found that the genetic predisposition of RLS leads to a decrease in serum ferritin, but the inverse proposition is not valid (decreased serum ferritin leading to RLS). Simultaneously, there is no evidence to support a potential causal relationship between peripheral iron deficiency and the onset of RLS or ADHD, but RLS has a genetic predisposition to reduced serum ferritin levels.

### 3.1 The causal relationship from ADHD to RLS

We performed MR analysis using the final 23 SNPs that were significantly and independently associated with ADHD and found that the genetic predisposition to ADHD was closely associated with an increased risk of RLS (OR = 1.20 (95%CI =1.08,1.34), p = 0.001[FDR=0.018]). The Weighted Median method (OR =1.21 (95%CI = 1.05,1.41), p = 0.01) yielded the same result, while MR-Egger regression (OR =1.27 (95%CI =0.69,2.35), p = 0.46) showed significant results in the opposite direction (**Table 2**). The MR Egger regression intercept was -0.0035 (p=0.8638), providing no evidence of unbalanced pleiotropy. No significant heterogeneity was found using the Cochran Q test in this study, and the MR-PRESSO global test result showed no detection of outlier SNPs (P>0.05). We used an instrument P threshold of 5x10e-8, and 23 SNPs were therefore used as instrumental tools, and the resulting IVW and Weighted Median method models still showed P>0.05. Visualization of sensitivity analysis showed that SNPs closely related to RLS and ADHD were stable in scatter plots; funnel plots showed that SNPs on both sides were evenly distributed and symmetrical, indicating no pleiotropy in MR analysis. At the same time, no single-nucleotide polymorphism had a major effect on the primary outcome of the study. Therefore, this study still uses the results of IVW as the main basis for judgment, and the genetic predisposition to ADHD can increase the risk of Restless Legs Syndrome.

**Table 2:** MR analysis between ADHD and RLS.

Exposure	Outcome	Nsnp	MR-Egger					Weighted Median					Inverse Variance Weightedver				
			OR	(95%CI)	Beta	SE	P value	OR	(95%CI)	Beta	SE	P value	OR	(95%CI)	Beta	SE	P value
**ADHD**	**RLS**	23	1.27	(-0.38,0.85)	0.24	0.31	0.46	1.21	(0.04,0.35)	0.19	0.08	0.01	1.20	(0.07,0.29)	0.18	0.06	0.001
**RLS**	**ADHD**	11	1.07	(-0.03,0.16)	0.07	0.05	0.22	1.06	(0.01,0.11)	0.06	0.03	0.01	1.04	(-0.01,0.09)	0.04	0.02	0.11
**RLS**	**Serum ferritin**	11	1.03	(-0.01,0.06)	0.03	0.02	0.18	1.02	(-0.00,0.04)	0.02	0.01	0.12	1.02	(-0.00,0.04)	0.02	0.01	0.047

MR, Mendelian randomization; IVW, Inverse Variance Weighted; CI, Confidence Interval.

### 3.2 The causal relationship from RLS to ADHD

Based on 11 significant and independent SNPs associated with RLS, no evidence of a potential causal effect of RLS on ADHD was found (OR = 1.04(95% CI = 0.99,1.09, p = 0.11[FDR=0.25]). The same result was also obtained using MR-Egger regression (OR = 1.07(95% CI =0.97,1.18), p = 0.22). The Weighted Median method yielded a significant result in the opposite direction (OR = 1.06(95% CI = 1.02,1.12), p = 0.01)(**Table 2**). However, the IVW result was used as the primary basis in our study.

### 3.3 The genetic correlation between ADHD and RLS

Linkage disequilibrium score regression (LDSC) was performed using GWAS summary data for ADHD and RLS to assess the genetic correlation between the two diseases. The results showed a lack of genetic correlation between RLS and ADHD (Rg = 0.3, SE = 0.16, P = 0.068).

### 3.4 The causal relationship between ADHD, RLS, and iron status

Based on the observed causal relationship between ADHD and RLS, as well as the clinical observation of the relationship between RLS, ADHD, and iron deficiency, and their response to iron therapy, we thought that the pathogenesis of RLS and ADHD may also be closely related to iron status. Therefore, we conducted an MR analysis on the causal relationship between iron status and RLS and ADHD. We still used the inverse variance weighted (IVW) method as the main MR analysis method to investigate the relationship between RLS and iron status (serum iron, ferritin, transferrin saturation, and total iron binding capacity), while treating iron status as exposure and RLS and ADHD as outcomes for reverse MR. At the same time, MR evidence supports a significant genetic risk of RLS causing a decrease in ferritin in the blood (OR = 1.02 (95% CI = -0.00,0.04), p = 0.047), but the inverse proposition is not valid (see **Supplementary**). There was no causal relationship between peripheral iron status (ferritin, transferrin saturation, and total iron binding capacity) and RLS or ADHD. The specific correlation analysis results are shown in the **Supplementary**.

## 4 Discussion

### 4.1 The summary of the result

The widespread application of summary-level statistics has gradually become the mainstream of causal inference methods, providing further reference for the study of the mechanisms of disease. This study used Mendelian randomization and Linkage disequilibrium score calculation to investigate the causal relationships between RLS and ADHD, as well as between iron status and both conditions. We found that genetic predisposition to ADHD increases the risk of developing RLS, but no evidence exists for the reverse causality or a genetic correlation between the two. Interestingly, while no direct causal relationship was found between peripheral iron levels and the pathogenesis of either ADHD or RLS, RLS genetic predisposition appears to confer a risk of lower serum ferritin levels.

### 4.2 The relationship between ADHD and RLS

Previous observational and meta-analysis studies have shown that ADHD has a high incidence in patients diagnosed with RLS (14, 16, 30, 31). Although the two often co-occur, the unclear temporal order leads to ambiguity in the causal relationship, and there are several inconsistent conclusions in current research. One multicenter double-blind clinical trial on dopaminergic treatment for restless legs syndrome (RLS) in children showed that levodopa significantly improved RLS/periodic limb movements during sleep (PLMS) but did not improve attention deficit hyperactivity disorder (ADHD) (32). A meta-analysis indicated the same perspective: there was a lack of correlation between RLS and ADHD symptoms (14). However according to another case report: a patient with both attention deficit hyperactivity disorder and restless leg syndrome showed significant improvement in symptoms after treatment with dopamine agonist ropinirole, indicating that RLS and ADHD may have a common pathogenesis in the dopamine regulation loop (33). A meta-analysis from 1990 to 2006 showed that patients with primary sleep disorders such as RLS may be misdiagnosed as attention deficit hyperactivity disorder and often appear as comorbidities (34). This is consistent with our research findings, it indicates that RLS is highly correlated with attention deficit hyperactivity disorder and may be related to common central nervous dopaminergic dysfunction. However, a clinical trial using variant genes (MEIS1, BTBD9, and MAP2K5) from two genome-wide association studies for exploratory analysis of genetic associations in 224 well-characterized family samples did not observe a genetic association between the two (35). This is consistent with our linkage disequilibrium score regression results. The lack of genetic correlation suggests that this causal relationship is not driven by genetic factors, but may be due to a biological link between ADHD and RLS or their exposure to the same environmental or lifestyle factors, for example: Education level, Sleep deprivation, and so on. Therefore, a more comprehensive consideration of various factors is needed to better understand their relationship. Although some studies expressed opposing views (36).

### 4.3 Two hypotheses between ADHD and RLS

There have been two hypotheses that support the association between RLS and ADHD for a long time, one hypothesis is the dopamine hypothesis. A case report using dopamine therapy showed after using dopamine agonists, the patient’s RLS and ADHD symptoms were significantly improved (37). Multiple observational studies and meta-analysis studies also suggest there may be a common disease defect in the dopamine loop between the two (14, 38). However, some studies have shown that dopamine treatment is only effective for a short time, and long-term use can further lead to postsynaptic downregulation and more severe restless leg syndrome (39), which has made us think more deeply about the relationship between the two. For example, Pa and RLS have a good therapeutic response to dopamine, but a Mendelian randomization study on parkinsonism and RLS also stated that there is no causal relationship between parkinsonism and RLS from a genetic perspective, and there is no genetic correlation (40).

The mainstream theory in clinical practice is that the lack of iron elements is the potential pathological mechanism of comorbidity between RLS and ADHD, and there are considerable studies in recent literature (35, 38, 41, 42). An animal model experiment supported the hypothesis that RLS iron deficiency behavior can produce RLS symptoms and found that giving effective dopaminergic treatment to RLS mice would also reduce the deterioration of RLS-like behavior (43). Clinical trial research found that 33% of adult patients with ADHD were diagnosed with RLS, and their frequency of iron deficiency was higher than that of the control group without RLS (17). At the same time, multiple studies have shown that supplementing iron helps treat ADHD (44–46). Therefore, both RLS and ADHD may have iron and dopamine deficiencies.

### 4.4 Proposing two new hypotheses on the relationship between ADHD and RLS

We think that although there is a common pathogenesis between the two, different pathological processes and molecular mechanisms are involved. Based on the above hypothesis, we think that the key link in the relationship between iron and dopamine may be the cause of the heterogeneity of the pathogenesis of restless leg syndrome and ADHD (47). Therefore, we also conducted a Two-sample MR on ADHD, RLS, and peripheral iron status (Four traits: ferritin, iron, total iron binding capacity, and transferrin saturation). However, the MR analysis results showed that peripheral iron status has no causal relationship with the onset of ADHD or RLS, which is consistent with the results of a clinical trial and also confirms RLS has a genetic predisposition to reduced serum ferritin levels. This is consistent with the conclusion that serum ferritin in RLS patients is usually low and negatively correlated with the severity of RLS symptoms (48). Through further analysis of the above phenomena, we draw the following inference: brain iron is a highly related influencing factor for RLS and ADHD. This may be due to the different regulatory mechanisms between peripheral iron status and brain iron. We only used summary statistics for peripheral iron status. However, so far, due to the existence of a hydrophobic barrier formed by the blood-brain barrier, the degree of correlation between serum iron and brain iron is not clear. Related research shows that the brain regulates brain iron by regulating the permeability of the blood-brain barrier. They have two different regulatory mechanisms (47, 49, 50).

According to existing research, brain iron is closely related to neurological diseases (49, 51, 52). In ADHD and RLS, the same is true. Magnetic resonance imaging studies have shown that the brain iron content in the thalamic region of ADHD patients is lower than that of normal healthy people (49, 53, 54), while the RLS cohort suggests a decrease in brain iron content in the substantia nigra, caudate nucleus, and temporal and occipital lobe compartments (55). There are also imaging-related studies that confirm this view (56, 57). A prospective large-scale ADHD magnetic resonance (MR) study showed that abnormal brain iron levels in attention deficit hyperactivity disorder reflect the involvement of iron in the dopamine metabolism pathway (58). A study based on diet-induced brain iron deficiency rodent models, human magnetic resonance imaging studies, autopsy materials, and other aspects showed that brain iron deficiency is the main factor causing RLS potential pathology (59). There are many studies on RLS and brain iron and dopamine, but the specific correlation mechanism between them has not been elucidated. In addition, iron is a cofactor of tyrosine hydroxylase, which is the rate-limiting enzyme for dopamine synthesis (47). Brain iron deficiency can lead to central dopamine synthesis disorders and functional disorders (59, 60). A meta-analysis showed that there is a strong coexistence between RLS and ADHD, which may be related to sleep fragmentation and damage to the dopamine system. Therefore, Brain iron deficiency plays an important role in the comorbidity of RLS and ADHD (14).

Another hypothesis proceeding from pathogenic mechanisms underlying attention-deficit/hyperactivity disorder (ADHD), we theorize chronic inflammation in ADHD may elicit restless legs syndrome (RLS) onset. Moreover, ADHD complicated by RLS likely constitutes preliminary pathology preceding secondary Tourette Syndrome (TS), the most severe ADHD comorbidity. Both TS and RLS manifest involuntarily hyperkinetic features, though differing symptomatically in severity (61). Relevant literature indicates pathophysiological commonalities across ADHD, TS, and RLS. ADHD pathogenesis proves highly correlated with chronic inflammation (62), which through iron regulatory cytokine production and secretion, may deplete intracellular iron stores, eliciting an iron-deficient state (63). Though peripheral iron levels appear unaffected, specialized central-peripheral iron regulation impacts cerebral iron availability. Moreover, cerebral function is exquisitely sensitive to substrate fluctuations (49), conferring susceptibility to RLS. Our investigations demonstrate RLS consumes serum transferrin, which could further impede cerebral iron repletion, exacerbating TS outcomes. Collectively, these observations situate RLS as a likely pathological precursor to secondary TS in ADHD, underscoring the critical need for timely therapeutic intervention among ADHD patients with RLS comorbidities.

Building on these hypothetical frameworks, our subsequent research aims to intensely investigate underpinnings linking ADHD, TS, and RLS pathology centered upon cerebral iron homeostasis, while additionally scrutinizing any mediating environmental/lifestyle influences through causal analytic approaches.

### 4.5 Limitations of the study and conclusions

Limitations of this article: Because this study uses summary statistics, and there is an overlap of symptoms among restless leg syndrome, Periodic limb movement disorder (PLMD), and ADHD, we cannot rule out patients with both diseases in the dataset, which will make the results clearer, but more detailed data is difficult to obtain. In addition, the GWAS summary statistics used in this study are from European population disease research, so our conclusions may not apply to other racial groups. More research from more populations may be needed to further study the relationship between RLS and ADHD. It is worth noting that Mendelian randomization can only explain the relationship between RLS, ADHD, and peripheral iron status at the genetic level. Perhaps due to the current limitations of GWAS technology, some genetic spatial structural variations that can promote comorbidity have not been detected.

By exploring the relationship between RLS, ADHD, and peripheral iron status from a genetic perspective through MR, our study confirmed the hypothesis that ADHD is an important risk factor for the onset of RLS, confirmed that the onset of the two is not related to peripheral iron status, and concluded that RLS may cause a decrease in serum iron, providing further evidence for related research. However, clinically, treatment cannot be given to a single disease alone, and multiple combined treatments may be a better choice. It is worth noting that for ADHD, it is necessary to carefully consider whether RLS is combined, especially for children.

The SNPs obtained by various methods such as correlation, independence, and removal of linkage disequilibrium in this study meet the hypothesis of effective instrumental variables, and various methods are consistent with the IVW conclusion and there is no pleiotropy disturbance.

## 5 Conclusion

Our research indicates that a hereditary inclination toward attention deficit hyperactivity disorder may augment the susceptibility to restless leg syndrome manifestation, but no genetic association exists between the two diseases. This causal relationship may not be caused by genetic factors but may be due to a biological link or exposure to the same environmental or lifestyle factors. At the same time, there is no evidence to support the existence of a potential causal relationship between peripheral iron deficiency and RLS or ADHD, but RLS can cause a decrease in serum ferritin. Future studies examining the interrelationship between ADHD and RLS will delve deeper into the mechanisms linking ADHD-inflammation-brain iron deficiency-RLS and ADHD-TS-RLS.

## Declaration of generative AI in scientific writing

During the preparation of this work, the author(s) used [Newbing] in order to polish sentences. After using this tool, the author(s) reviewed and edited the content as needed and take(s) full responsibility for the content of the publication.

## Data availability statement

The original contributions presented in the study are included in the article/**Supplementary Material**. Further inquiries can be directed to the corresponding author.

## Ethics statement

We used publicly available GWAS summary statistics and each GWAS was approved by its corresponding ethics committee and followed the tenants of the Declaration of Helsinki. The studies were conducted in accordance with the local legislation and institutional requirements. The human samples used in this study were acquired from gifted from another research group. Written informed consent to participate in this study was not required from the participants or the participants’ legal guardians/next of kin in accordance with the national legislation and the institutional requirements.

## Author contributions

GX: Conceptualization, Methodology, Project administration, Writing – original draft. HS: Formal analysis, Resources, Writing – review & editing. QL: Writing – original draft, Data curation, Validation, Writing – review & editing. JH: Formal analysis, Writing – review & editing. JG: Writing – review & editing. ZL: Writing – review & editing. CZ: Writing – review & editing. ZH: Writing – review & editing. YC: Formal analysis, Funding acquisition, Writing – review & editing. BZ: Funding acquisition, Methodology, Supervision, Writing – review & editing.
