# Supplementary material for: Association among attention-deficit hyperactivity disorder, restless legs syndrome, and peripheral iron status: a two-sample Mendelian randomization study
Source: Front Psychiatry. 2024 May 8;15:1310259. doi: 10.3389/fpsyt.2024.1310259 (PMC11109751; doi:10.3389/fpsyt.2024.1310259)
Supplement: Supplementary file 2 [file Presentation_1.pdf]

Exposure	Outcome	NSNP	MR-Egger					Weighted Median					IVW				
			OR	(95%CI)	Beta	SE	P value	OR	(95%CI)	Beta	SE	P value	OR	(95%CI)	Beta	SE	P value
ADHD	RLS	23	1.27	(-0.38,0.85)	0.24	0.31	0.46	1.21	(0.04,0.35)	0.19	0.08	0.01	1.20	(0.07,0.29)	0.18	0.06	0.001
RLS	ADHD	11	1.07	(-0.03,0.16)	0.07	0.05	0.22	1.06	(0.01,0.11)	0.06	0.03	0.01	1.04	(-0.01,0.09)	0.04	0.02	0.11
ADHD	Ironferritin	24	1.03	(-0.06,0.13)	0.03	0.05	0.51	0.98	(-0.06,0.01)	-0.02	0.02	0.18	0.99	(-0.04,0.01)	-0.01	0.01	0.23
	Iron	25	1.00	(-0.13,0.12)	-0.01	0.06	0.94	1.00	(-0.04,0.03)	-0.01	0.02	0.81	1.00	(-0.04,0.02)	-0.01	0.02	0.42
	Irontsat	25	1.00	(-0.06,0.02)	-0.03	0.06	0.59	0.98	(-0.06,0.02)	-0.02	0.02	0.35	1.00	(-0.45,0.02)	-0.02	0.02	0.34
	Irontibc	25	0.95	(-0.18,0.08)	-0.05	0.06	0.44	1.04	(-0.01,0.08)	0.04	0.02	0.10	0.99	(-0.01,0.05)	0.02	0.02	0.15
RLS	Ironferritin	11	1.03	(-0.01,0.06)	0.03	0.02	0.18	1.02	(-0.00,0.04)	0.02	0.01	0.12	1.02	(-0.00,0.04)	0.02	0.01	0.047
	Iron	10	0.99	(-0.10,0.09)	-0.01	0.05	0.88	1.01	(-0.20,0.04)	0.01	0.02	0.52	0.99	(-0.01,0.04)	0.01	0.01	0.22
	Irontsat	9	0.94	(-0.16,0.04)	-0.06	0.05	0.30	1.02	(-0.01,0.05)	0.02	0.02	0.28	1.00	(0.00,0.05)	0.02	0.01	0.09
	Irontibc	12	1.02	(-0.02,0.06)	0.02	0.02	0.26	1.02	(0.00,0.05)	0.02	0.01	0.05	1.02	(0.00,0.04)	0.02	0.01	0.08
Ironferritin	ADHD	52	0.98	(-0.19,0.15)	-0.02	0.09	0.84	0.95	(-0.18,008)	-0.02	0.05	0.43	0.98	(-0.11,0.07)	-0.02	0.05	0.73
	RLS	49	0.82	(-0.49,0.09)	-0.20	0.15	0.18	0.78	(-0.46,-0.03)	-0.24	0.11	0.03	0.88	(-0.28,0.03)	-0.13	0.08	0.11
Iron	ADHD	22	1.01	(-0.10,0.13)	0.01	0.06	0.83	0.98	(-0.12,0.08)	-0.02	0.05	0.74	0.96	(-0.12,0.04)	-0.04	0.04	0.29
	RLS	27	0.89	(-0.30,0.07)	-0.11	0.10	0.25	0.91	(-0.27,0.07)	-0.10	0.09	0.27	0.90	(-0.23,0.02)	-0.11	0.06	0.10
Irontsat	ADHD	23	1.03	(-0.07,0.13)	0.03	0.05	0.57	1.01	(-0.08,0.09)	0.01	0.04	0.86	0.92	(-0.09,0.04)	-0.02	0.03	0.48
	RLS	24	0.88	(-0.30,0.04)	-0.13	0.09	0.14	0.90	(-0.24,0.03)	-0.10	0.07	0.14	0.90	(-0.21,0.00)	-0.10	0.05	0.052
Irontibc	ADHD	25	0.99	(-0.12,0.09)	-0.01	0.05	0.84	0.97	(-0.12,0.07)	-0.03	0.05	0.61	0.99	(-0.09,0.06)	-0.01	0.04	0.69

	RLS	19	1.26	(-0.26,0.72)	0.23	0.25	0.36	1.11	(-0.17,0.37)	0.10	0.14	0.47	1.13	(-0.07,0.31)	0.12	0.10	0.22

Table 3 MR estimates from different methods of assessing the causal effect of.（after removing outliers）

Exposure	Outcome	IVW		MR-Egger	
		Cochran's Q	Q-P value	Cochran's Q	Q-P value
ADHD	RLS	24.17	0.34	24.14	0.29
RLS	ADHD	17.50	0.06	16.76	0.05
ADHD	Ironferritin	25.54	0.32	24.45	0.32
	Iron	31.90	0.13	31.88	0.10
	Irontsat	20.97	0.64	20.87	0.59
	Irontibc	20.61	0.66	19.23	0.69
RLS	Ironferritin	16.35	0.09	15.85	0.07
	Iron	6.27	0.71	6.06	0.64
	Irontsat	4.91	0.77	2.36	0.94
	Irontibc	13.09	0.29	12.92	0.23
Ironferritin	ADHD	54.46	0.34	54.46	0.31
	RLS	58.77	0.14	58.34	0.12
Iron	ADHD	16.43	0.75	14.87	0.78
	RLS	13.41	0.86	13.40	0.82
Irontsat	ADHD	16.49	0.79	14.74	0.84
	RLS	17.50	0.78	17.32	0.75
Irontibc	ADHD	12.60	0.96	12.59	0.96
	RLS	14.55	0.69	14.31	0.65

Table 4 Heterogeneity tests(the Q-p values of the IVW and MR–Egger were both greater than 0.05).（after removing outliers）

Exposure	Outcome	Egger-intercept	Intercept-P value
ADHD	RLS	-0.0035	0.8638
RLS	ADHD	-0.0055	0.5449
ADHD	Ironferritin	-0.0033	0.3328
	Iron	-0.0005	0.9028
	Irontsat	0.0013	0.7545
	Irontibc	0.0051	0.2515
RLS	Ironferritin	-0.0016	0.6071
	Iron	0.0061	0.6589
	Irontsat	0.0110	0.1544
	Irontibc	-0.0012	0.7224
Ironferritin	ADHD	0.0001	0.9847
	RLS	0.0032	0.5605
Iron	ADHD	-0.0050	0.2260
	RLS	0.0006	0.9271
Irontsat	ADHD	-0.0053	0.1990
	RLS	0.0027	0.6769
Irontibc	ADHD	-0.0004	0.9265
	RLS	-0.0059	0.6307

Table 5 MR-PRESSO analysis and MR-Egger intercept(MR–Egger intercept p value > 0.05).（after removing outliers）

MR Presso	
ADHD to RLS	0.365
RLS to ADHD	0.227
ADHD to IP	0.688
ADHD to Iron	0.133
ADHD to Tsat	0.651
ADHD to Tibc	0.673
RLS to IP	0.153
RLS to Iron	0.644
RLS to Tsat	0.796
RLS to Tibc	0.500
IP to ADHD	0.385
IP to RLS	0.075
Iron to ADHD	0.767
Iron to RLS	0.943
Tsat to ADHD	0.813
Tsat to RLS	0.762
Tibc to ADHD	0.938
Tibc to RLS	0.335

Table 6 All MR Presso（after removing outliers）.
