# Supplementary material for: Association among attention-deficit hyperactivity disorder, restless legs syndrome, and peripheral iron status: a two-sample Mendelian randomization study
Source: Front Psychiatry. 2024 May 8;15:1310259. doi: 10.3389/fpsyt.2024.1310259 (PMC11109751; doi:10.3389/fpsyt.2024.1310259)
Supplement: Supplementary file 3 [file Presentation_2.pdf]

All Forest Plot

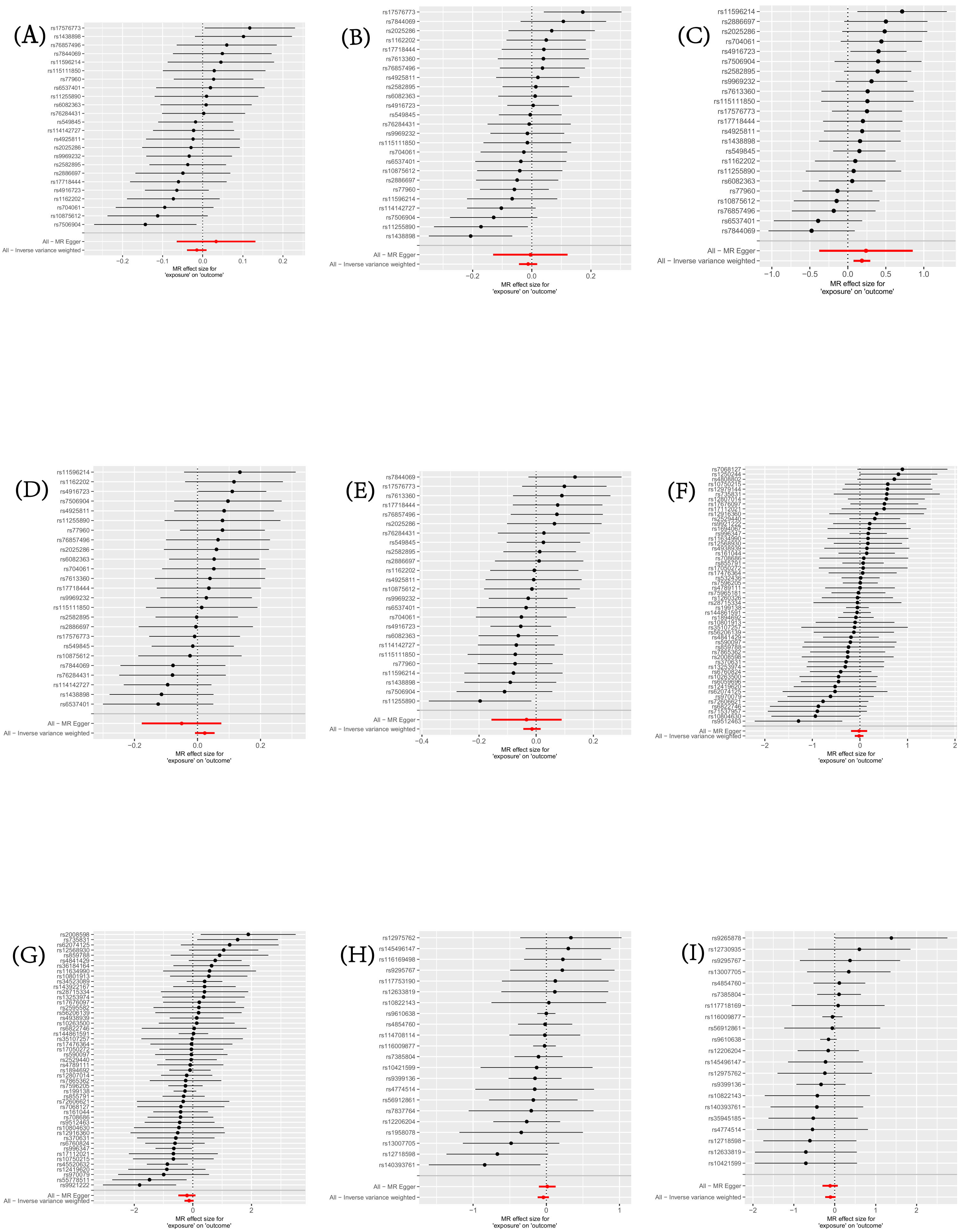

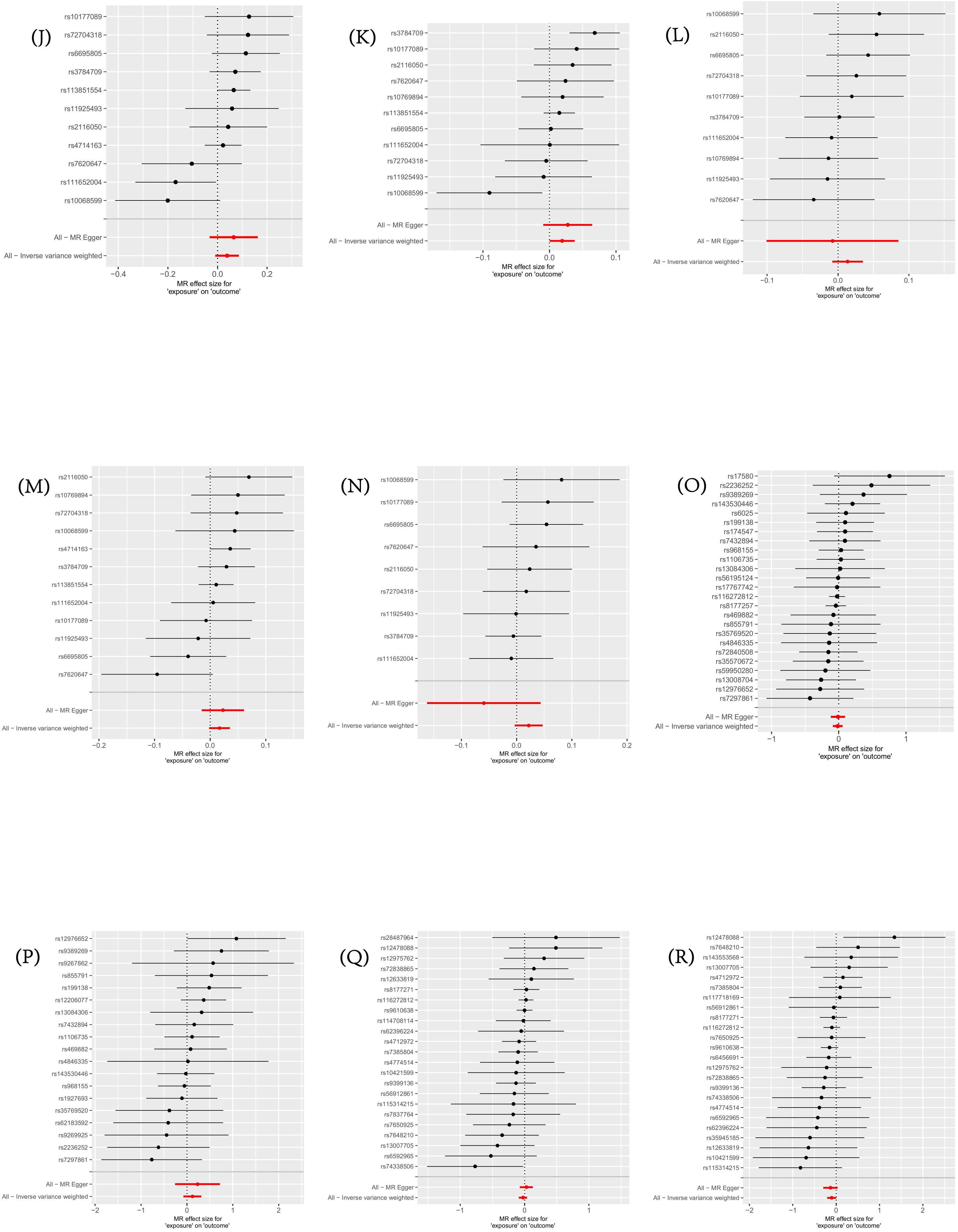
(J)
rs10177089
rs72704318
rs6695805
rs3784709
rs113851554
rs11925493
rs2116050
rs4714163
rs7620647
rs111652004
rs10068599
All − MR Egger
All − Inverse variance weighted
−0.4
−0.2
0.0
0.2
MR effect size for
'exposure' on 'outcome'
(K)
rs3784709
rs10177089
rs2116050
rs7620647
rs10769894
rs113851554
rs6695805
rs111652004
rs72704318
rs11925493
rs10068599
All − MR Egger
All − Inverse variance weighted
−0.1
0.0
0.1
MR effect size for
'exposure' on 'outcome'
(L)
rs10068599
rs2116050
rs6695805
rs72704318
rs10177089
rs3784709
rs111652004
rs10769894
rs11925493
rs7620647
All − MR Egger
All − Inverse variance weighted
−0.1
0.0
0.1
MR effect size for
'exposure' on 'outcome'
(M)
rs2116050
rs10769894
rs72704318
rs10068599
rs4714163
rs3784709
rs113851554
rs111652004
rs10177089
rs11925493
rs6695805
rs7620647
All − MR Egger
All − Inverse variance weighted
−0.2
−0.1
0.0
0.1
MR effect size for
'exposure' on 'outcome'
(N)
rs10068599
rs10177089
rs6695805
rs7620647
rs2116050
rs72704318
rs11925493
rs3784709
rs111652004
All − MR Egger
All − Inverse variance weighted
−0.1
0.0
0.1
0.2
MR effect size for
'exposure' on 'outcome'
(O)
rs17580
rs2236252
rs9389269
rs143530446
rs6025
rs199138
rs174547
rs7432894
rs968155
rs1106735
rs13084306
rs56195124
rs17767742
rs116272812
rs8177257
rs469882
rs855791
rs35769520
rs4846335
rs72840508
rs35570672
rs59950280
rs13008704
rs12976652
rs7297861
All − MR Egger
All − Inverse variance weighted
−1
0
1
MR effect size for
'exposure' on 'outcome'
(P)
rs12976652
rs9389269
rs9267862
rs855791
rs199138
rs12206077
rs13084306
rs7432894
rs1106735
rs469882
rs4846335
rs143530446
rs968155
rs1927693
rs35769520
rs62183592
rs9269925
rs2236252
rs7297861
All − MR Egger
All − Inverse variance weighted
−2
−1
0
1
2
MR effect size for
'exposure' on 'outcome'
(Q)
rs28487964
rs12478088
rs12975762
rs72838865
rs12633819
rs8177271
rs116272812
rs9610638
rs114708114
rs62396224
rs4712972
rs7385804
rs4774514
rs10421599
rs9399136
rs56912861
rs115314215
rs7837764
rs7650925
rs7648210
rs13007705
rs6592965
rs74338506
All − MR Egger
All − Inverse variance weighted
−1
0
1
MR effect size for
'exposure' on 'outcome'
(R)
rs12478088
rs7648210
rs143553568
rs13007705
rs4712972
rs7385804
rs117718169
rs56912861
rs8177271
rs116272812
rs7650925
rs9610638
rs6456691
rs12975762
rs72838865
rs9399136
rs74338506
rs4774514
rs6592965
rs62396224
rs35945185
rs12633819
rs10421599
rs115314215
All − MR Egger
All − Inverse variance weighted
−2
−1
0
1
2
MR effect size for
'exposure' on 'outcome'

Figure 2 The forest plots of the association between genetic predicted ADHD, RLS, and Iron status （after removing outliers）

Note: * using a relaxed instrument threshold (P< 1×10-8)

A:ADHD to Ironferritin;
B:ADHD to Iron;
C:ADHD to RLS;
D:ADHD to Tibc;
E:ADHD to Tsat;
F:Ironferritin to ADHD;
G:Ironferritin to RLS;
H:Iron to ADHD;
I:Iron to RLS;
J:RLS to ADHD;
K:RLS to ADHD;
L:RLS to Ironferritin;
M:RLS to Tibc;
N:RLS to Tsat;
O:Tibc to ADHD;
P:Tibc to RLS;
Q:Tsat to ADHD;
R:Tsat to RLS

Notes:
ADHD:Attention Deficit Hyperactivity Disorder
RLS:Restless Legs Syndrome
Tsat:Irontsat
Tibc:Irontibc

All Funnel Plot

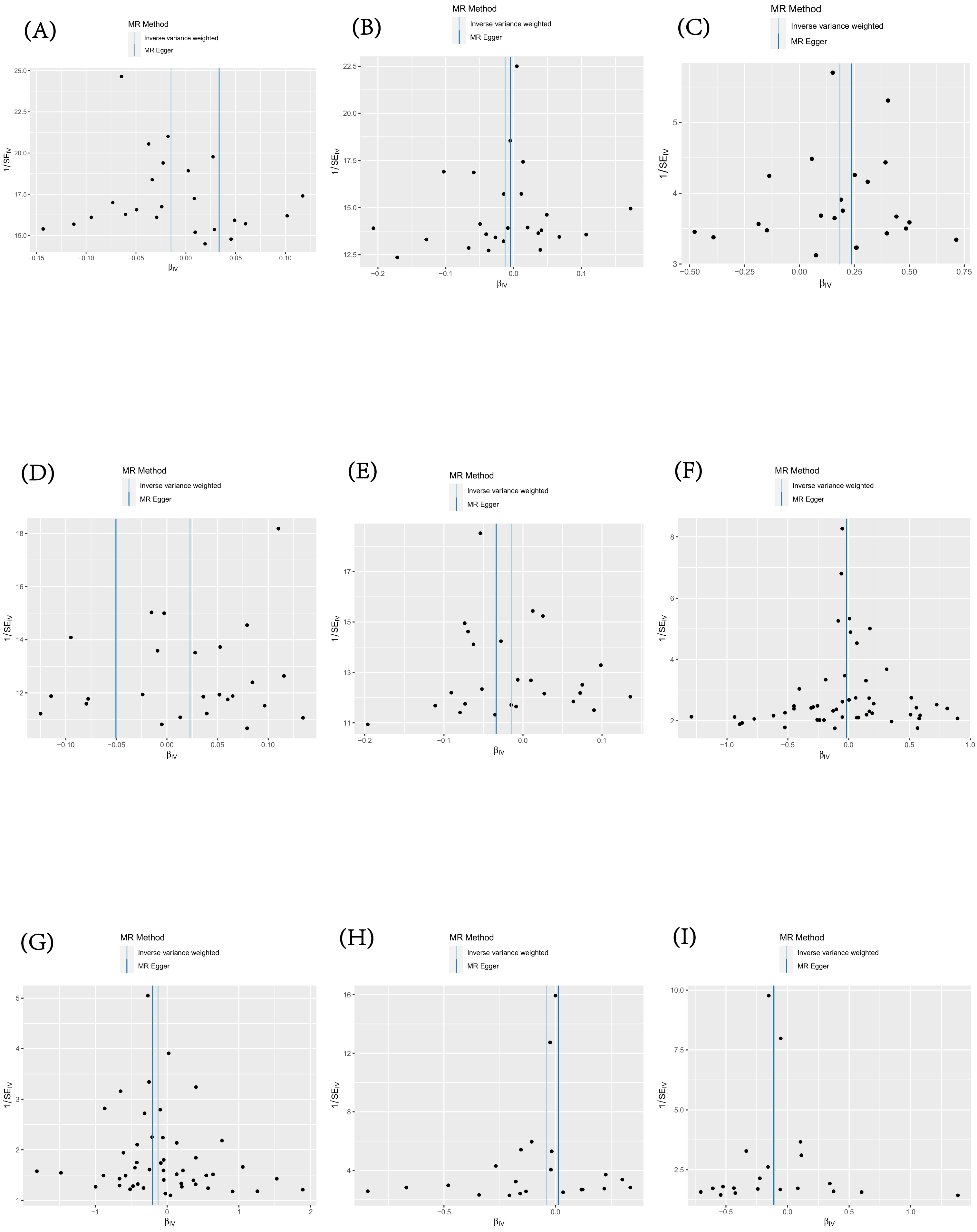

(J)

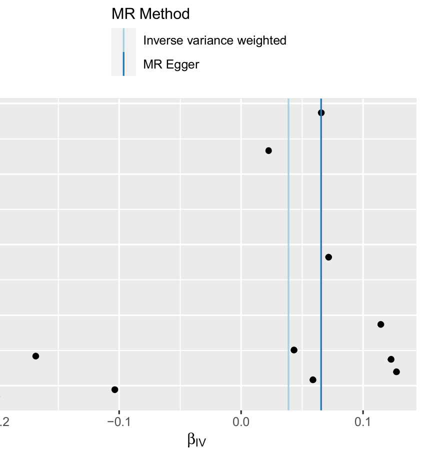

MR Method
Inverse variance weighted
MR Egger
$1/SE_{IV}$
β_{IV}

(K)

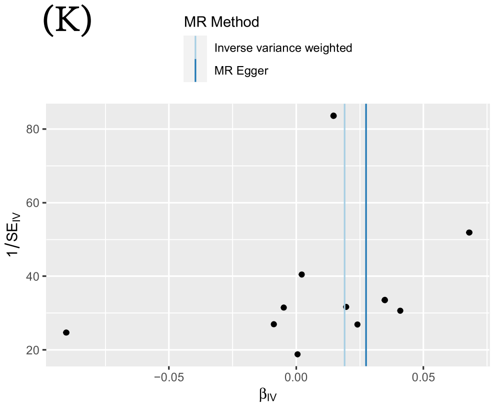

MR Method
Inverse variance weighted
MR Egger
$1/SE_{IV}$
β_{IV}

(L)

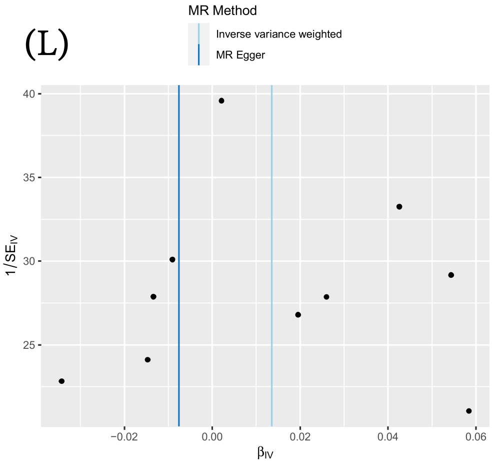

MR Method
Inverse variance weighted
MR Egger
$1/SE_{IV}$
β_{IV}

(M)

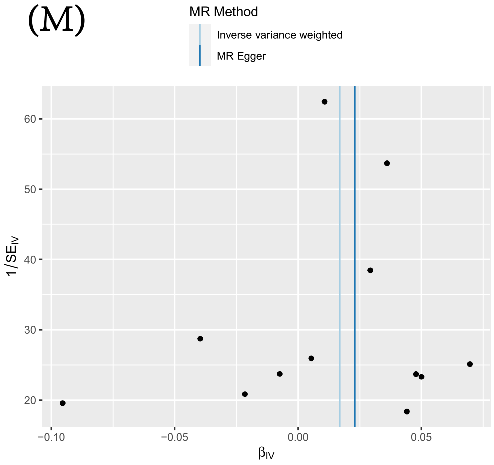

MR Method
Inverse variance weighted
MR Egger
$1/SE_{IV}$
β_{IV}

(N)

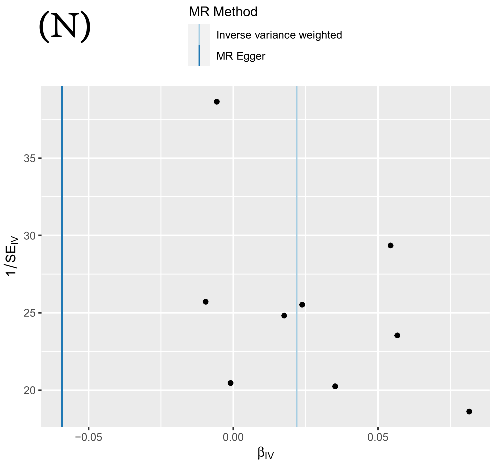

MR Method
Inverse variance weighted
MR Egger
$1/SE_{IV}$
β_{IV}

(O)

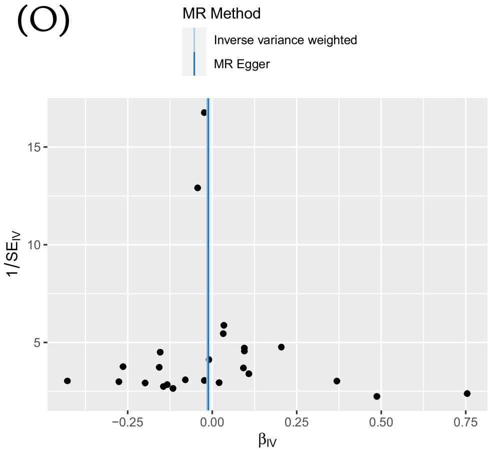

MR Method
Inverse variance weighted
MR Egger
$1/SE_{IV}$
β_{IV}

(P)

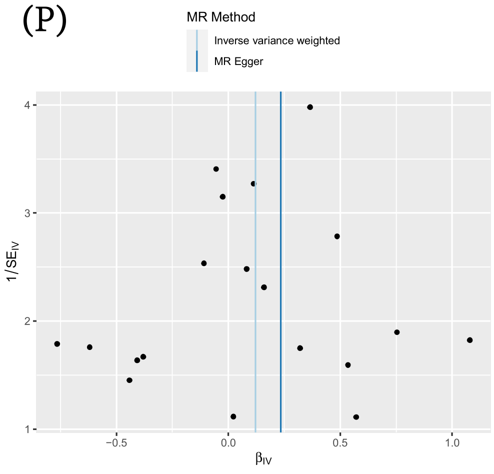

MR Method
Inverse variance weighted
MR Egger
$1/SE_{IV}$
β_{IV}

(Q)

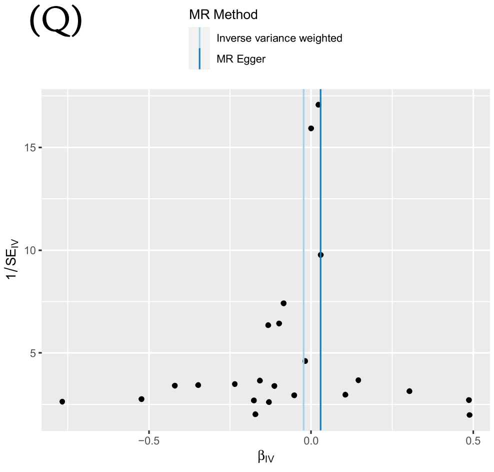

MR Method
Inverse variance weighted
MR Egger
$1/SE_{IV}$
β_{IV}

(R)

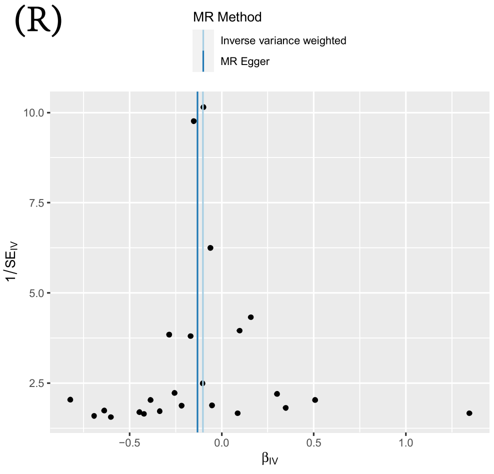

MR Method
Inverse variance weighted
MR Egger
$1/SE_{IV}$
β_{IV}

Figure 3 The funnel plots of ADHD, RLS, and Iron status （after removing outliers）

A:ADHD to Ironferritin;
B:ADHD to Iron;
C:ADHD to RLS;
D:ADHD to Tibc;
E:ADHD to Tsat;
F:Ironferritin to ADHD;
G:Ironferritin to RLS;
H:Iron to ADHD;
I:Iron to RLS;
J:RLS to ADHD;
K:RLS to ADHD;
L:RLS to Ironferritin;
M:RLS to Tibc;
N:RLS to Tsat;
O:Tibc to ADHD;
P:Tibc to RLS;
Q:Tsat to ADHD;
R:Tsat to RLS

Notes:
ADHD:Attention Deficit Hyperactivity Disorder
RLS:Restless Legs Syndrome
Tsat:Irontsat
Tibc:Irontibc

All Leave-One-Out

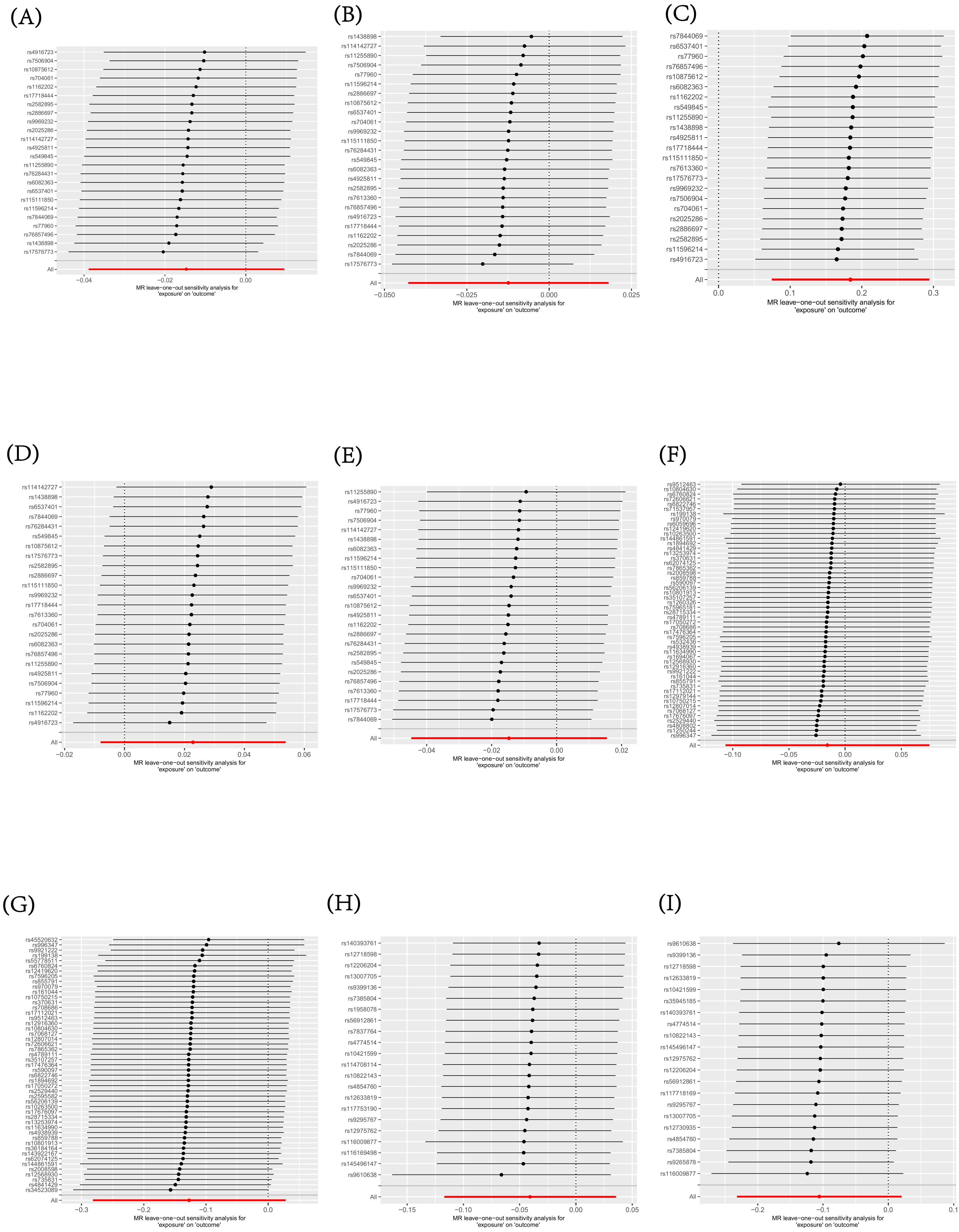

(J)

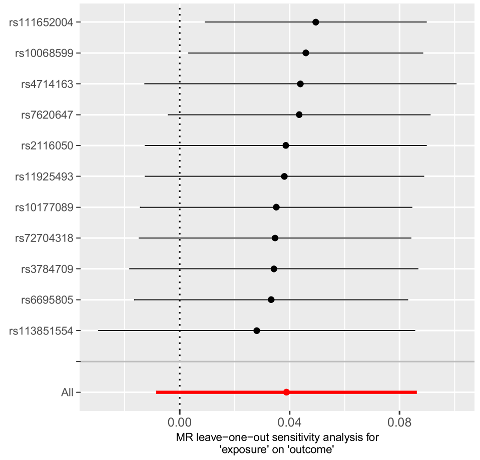
rs111652004
rs10068599
rs4714163
rs7620647
rs2116050
rs11925493
rs10177089
rs72704318
rs3784709
rs6695805
rs113851554
All
0.00
0.04
0.08
MR leave−one−out sensitivity analysis for
'exposure' on 'outcome'

(K)

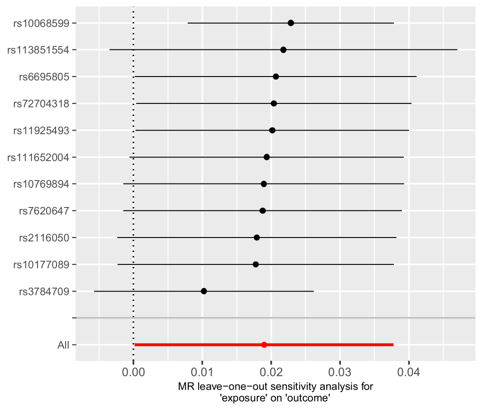
rs10068599
rs113851554
rs6695805
rs72704318
rs11925493
rs111652004
rs10769894
rs7620647
rs2116050
rs10177089
rs3784709
All
0.00
0.01
0.02
0.03
0.04
MR leave−one−out sensitivity analysis for
'exposure' on 'outcome'

(L)

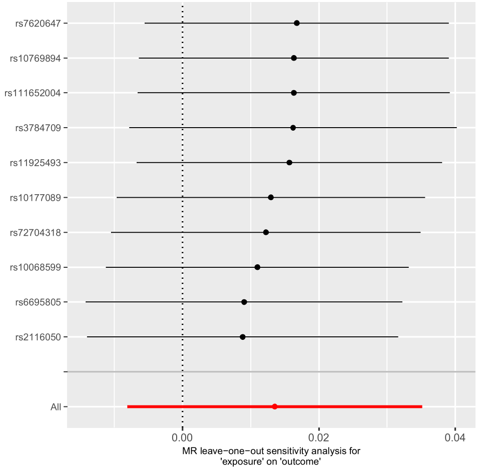
rs7620647
rs10769894
rs111652004
rs3784709
rs11925493
rs10177089
rs72704318
rs10068599
rs6695805
rs2116050
All
0.00
0.02
0.04
MR leave−one−out sensitivity analysis for
'exposure' on 'outcome'

(M)

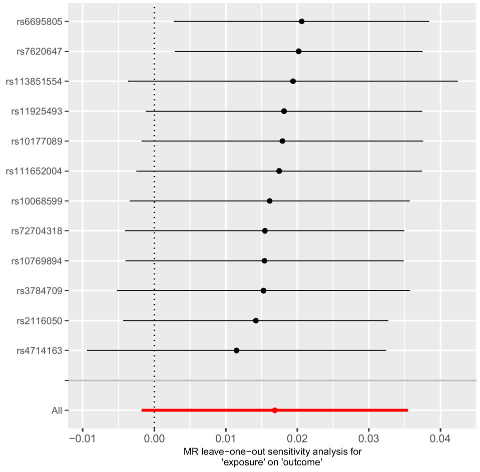
rs6695805
rs7620647
rs113851554
rs11925493
rs10177089
rs111652004
rs10068599
rs72704318
rs10769894
rs3784709
rs2116050
rs4714163
All
−0.01
0.00
0.01
0.02
0.03
0.04
MR leave−one−out sensitivity analysis for
'exposure' on 'outcome'

(N)

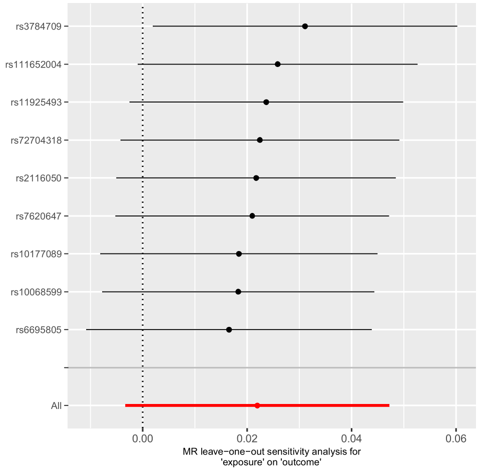
rs3784709
rs111652004
rs11925493
rs72704318
rs2116050
rs7620647
rs10177089
rs10068599
rs6695805
All
0.00
0.02
0.04
0.06
MR leave−one−out sensitivity analysis for
'exposure' on 'outcome'

(O)

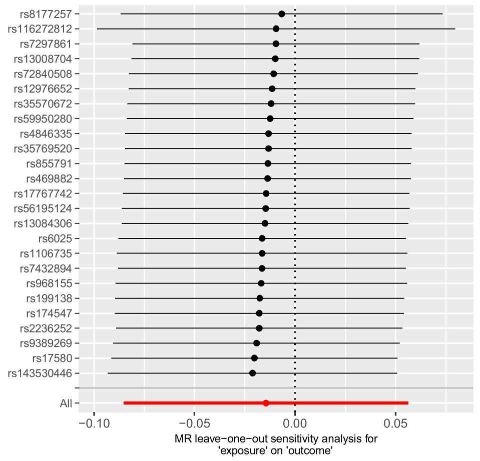
rs8177257
rs116272812
rs7297861
rs13008704
rs72840508
rs12976652
rs35570672
rs59950280
rs4846335
rs35769520
rs855791
rs469882
rs17767742
rs56195124
rs13084306
rs6025
rs1106735
rs7432894
rs968155
rs199138
rs174547
rs2236252
rs9389269
rs17580
rs143530446
All
−0.10
−0.05
0.00
0.05
MR leave−one−out sensitivity analysis for
'exposure' on 'outcome'

(P)

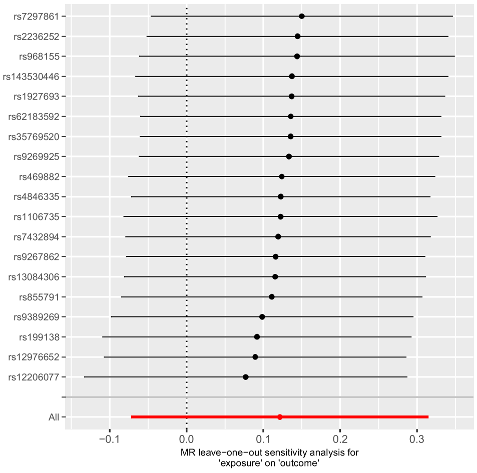
rs7297861
rs2236252
rs968155
rs143530446
rs1927693
rs62183592
rs35769520
rs9269925
rs469882
rs4846335
rs1106735
rs7432894
rs9267862
rs13084306
rs855791
rs9389269
rs199138
rs12976652
rs12206077
All
−0.1
0.0
0.1
0.2
0.3
MR leave−one−out sensitivity analysis for
'exposure' on 'outcome'

(Q)

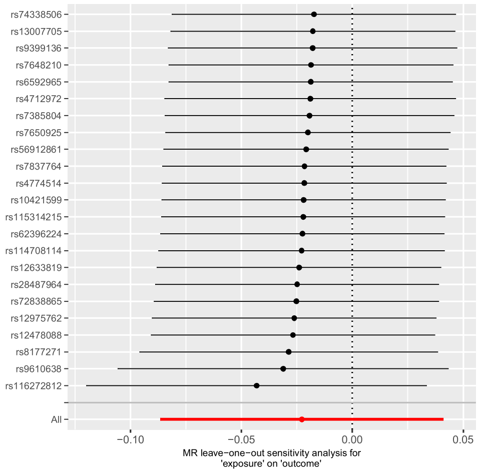
rs74338506
rs13007705
rs9399136
rs7648210
rs6592965
rs4712972
rs7385804
rs7650925
rs56912861
rs7837764
rs4774514
rs10421599
rs115314215
rs62396224
rs114708114
rs12633819
rs28487964
rs72838865
rs12975762
rs12478088
rs8177271
rs9610638
rs116272812
All
−0.10
−0.05
0.00
0.05
MR leave−one−out sensitivity analysis for
'exposure' on 'outcome'

(R)

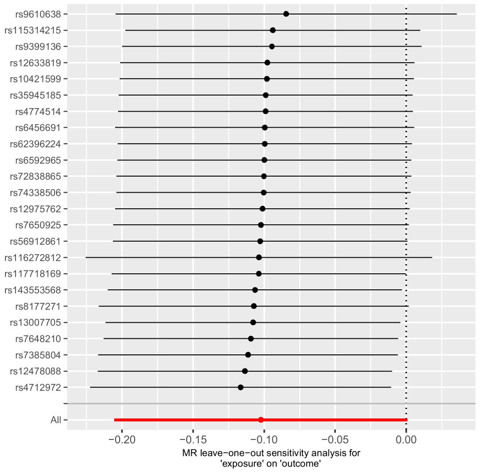
rs9610638
rs115314215
rs9399136
rs12633819
rs10421599
rs35945185
rs4774514
rs6456691
rs62396224
rs6592965
rs72838865
rs74338506
rs12975762
rs7650925
rs56912861
rs116272812
rs117718169
rs143553568
rs8177271
rs13007705
rs7648210
rs7385804
rs12478088
rs4712972
All
−0.20
−0.15
−0.10
−0.05
0.00
MR leave−one−out sensitivity analysis for
'exposure' on 'outcome'

Figure 4 Leave-one-out analyses for SNPs associated with ADHD, RLS, and Iron status （after removing outliers）
Note: * using a relaxed instrument threshold (P< 1×10-8).

A:ADHD to Ironferritin;
B:ADHD to Iron;
C:ADHD to RLS;
D:ADHD to Tibc;
E:ADHD to Tsat;
F:Ironferritin to ADHD;
G:Ironferritin to RLS;
H:Iron to ADHD;
I:Iron to RLS;
J:RLS to ADHD;
K:RLS to ADHD;
L:RLS to Ironferritin;
M:RLS to Tibc;
N:RLS to Tsat;
O:Tibc to ADHD;
P:Tibc to RLS;
Q:Tsat to ADHD;
R:Tsat to RLS
Notes:
ADHD:Attention Deficit Hyperactivity Disorder RLS:Restless Legs Syndrome
Tsat:Irontsat
Tibc:Irontibc

All Scatter Plot

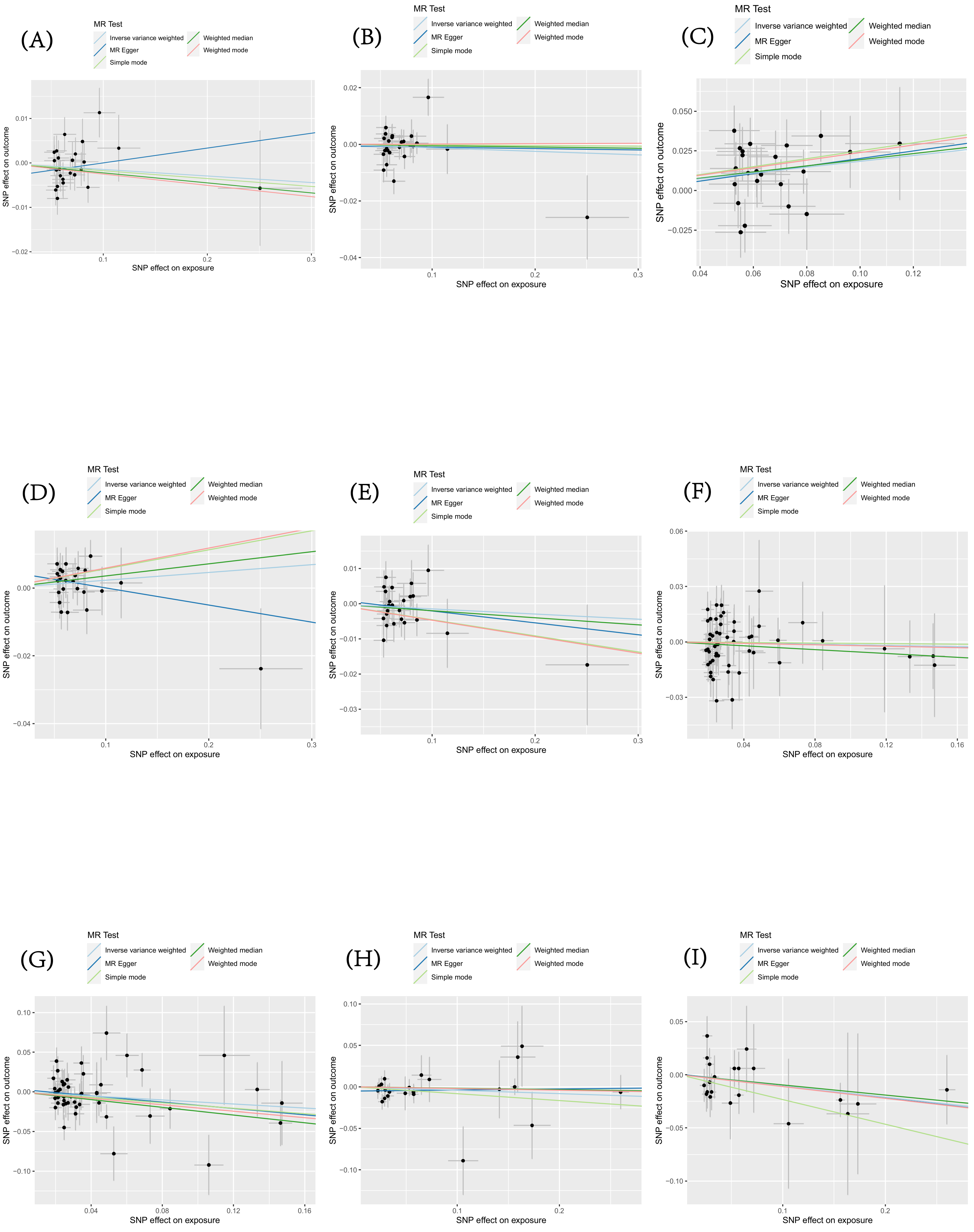

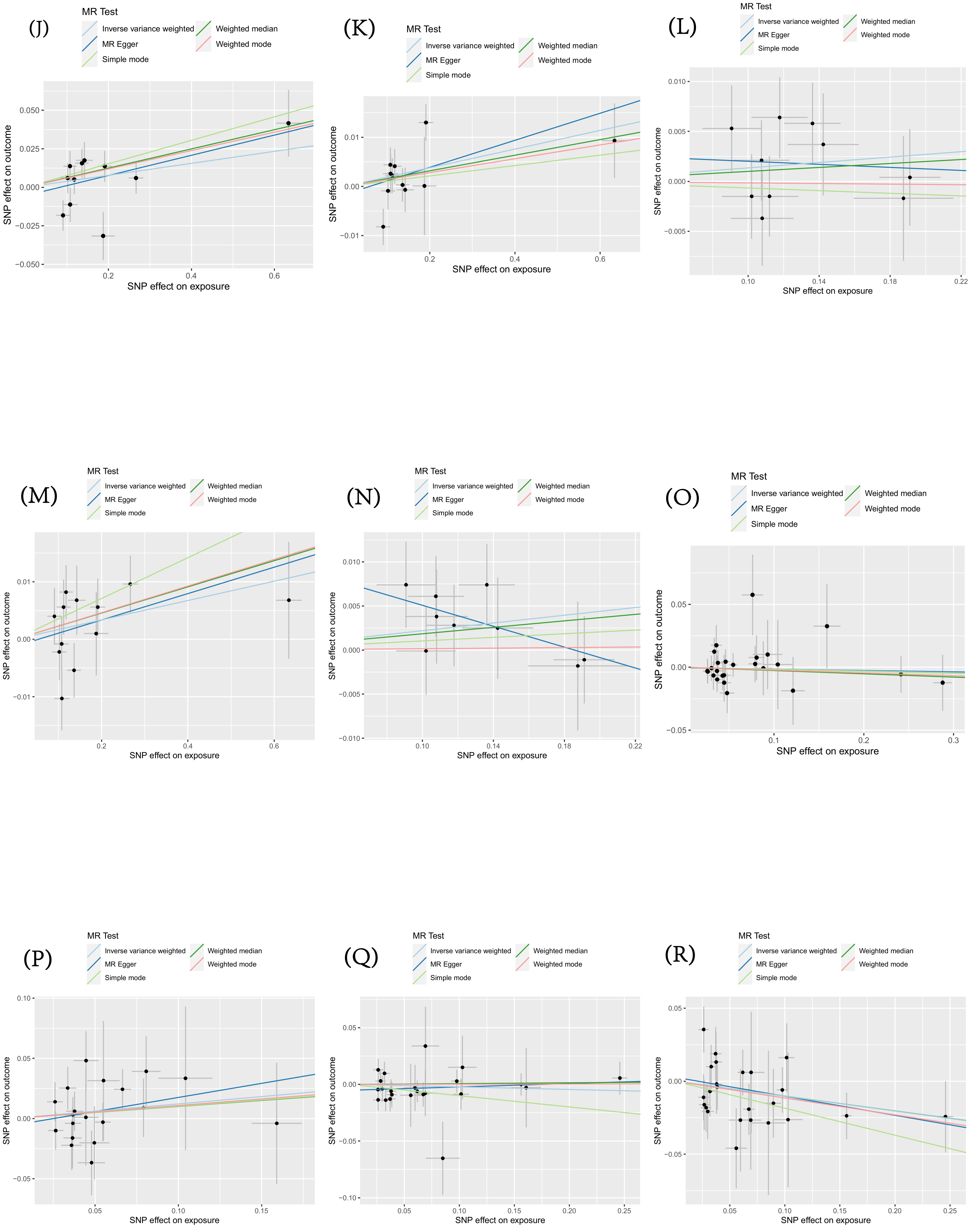
(J)
MR Test
Inverse variance weighted
MR Egger
Simple mode
Weighted median
Weighted mode
SNP effect on outcome
SNP effect on exposure
(K)
MR Test
Inverse variance weighted
MR Egger
Simple mode
Weighted median
Weighted mode
SNP effect on outcome
SNP effect on exposure
(L)
MR Test
Inverse variance weighted
MR Egger
Simple mode
Weighted median
Weighted mode
SNP effect on outcome
SNP effect on exposure
(M)
MR Test
Inverse variance weighted
MR Egger
Simple mode
Weighted median
Weighted mode
SNP effect on outcome
SNP effect on exposure
(N)
MR Test
Inverse variance weighted
MR Egger
Simple mode
Weighted median
Weighted mode
SNP effect on outcome
SNP effect on exposure
(O)
MR Test
Inverse variance weighted
MR Egger
Simple mode
Weighted median
Weighted mode
SNP effect on outcome
SNP effect on exposure
(P)
MR Test
Inverse variance weighted
MR Egger
Simple mode
Weighted median
Weighted mode
SNP effect on outcome
SNP effect on exposure
(Q)
MR Test
Inverse variance weighted
MR Egger
Simple mode
Weighted median
Weighted mode
SNP effect on outcome
SNP effect on exposure
(R)
MR Test
Inverse variance weighted
MR Egger
Simple mode
Weighted median
Weighted mode
SNP effect on outcome
SNP effect on exposure

Figure 5 The scatter plot of SNP effects on ADHD, RLS, and Iron status （after removing outliers）

Note: The slope of each line was corresponding to the estimated MR effect per method. The data are expressed as raw β values with 95% confidence interval. * Using a relaxed instrument threshold (P< 1×10-8).

A:ADHD to Ironferritin;
B:ADHD to Iron;
C:ADHD to RLS;
D:ADHD to Tibc;
E:ADHD to Tsat;
F:Ironferritin to ADHD;
G:Ironferritin to RLS;
H:Iron to ADHD;
I:Iron to RLS;
J:RLS to ADHD;
K:RLS to ADHD;
L:RLS to Ironferritin;
M:RLS to Tibc;
N:RLS to Tsat;
O:Tibc to ADHD;
P:Tibc to RLS;
Q:Tsat to ADHD;
R:Tsat to RLS

Notes:
ADHD:Attention Deficit Hyperactivity Disorder
RLS:Restless Legs Syndrome
Tsat:Irontsat
Tibc:Irontibc
